# Case report: *ETS1* gene deletion associated with a low number of recent thymic emigrants in three patients with Jacobsen syndrome

**DOI:** 10.3389/fimmu.2022.867206

**Published:** 2022-10-21

**Authors:** Tina Trachsel, Seraina Prader, Katharina Steindl, Jana Pachlopnik Schmid

**Affiliations:** ^1^ Division of Immunology, University Children’s Hospital Zurich, Zurich, Switzerland; ^2^ Institute of Medical Genetics, University of Zurich, Schlieren, Switzerland; ^3^ Pediatric Immunology, University of Zurich, Zurich, Switzerland

**Keywords:** Jacobsen syndrome, genetic disorder, ETS1, immunodeficiency, recent thymic emigrants

## Abstract

Jacobsen syndrome is a rare genetic disorder associated with a terminal deletion in chromosome 11. The clinical presentation is variable. Although immunodeficiency has been described in patients with Jacobsen syndrome, a clear genotype-phenotype correlation has not yet been established. Here, we report on the immunologic phenotypes of four patients with Jacobsen syndrome. All four patients showed one or more atypical immunologic features. One patient suffered from recurrent viral infections, two patients had experienced a severe bacterial infection and one had received antibiotic prophylaxis since early childhood. One patient had experienced severe, transient immune dysregulation. Hypogammaglobulinemia and low B cell counts were found in two patients, while the number of recent thymic emigrants (CD31+CD45RA+ CD4 cells) was abnormally low in three. When considering the six immune-related genes located within the affected part of chromosome 11 (*ETS1, TIRAP, FLI1, NFRKB, THYN1*, and *SNX19*), only the *ETS1* gene was found be deleted in the three patients with low numbers of recent thymic emigrants and non-switched memory B cells. Our findings support the hypothesis whereby Jacobsen syndrome is associated with a combined immunodeficiency with variable presentation. Further investigations of potential genotype-phenotype correlations are warranted and might help to personalize patient management in individuals lacking immune-related genes. In addition, we recommend immunologic follow-up for all patients with Jacobsen syndrome, as immune abnormalities may develop over time.

## Introduction

Jacobsen syndrome was first described in 1973 by the Danish geneticist Petra Jacobsen (1). This rare genetic disorder is associated with partial deletion of the distal arm of chromosome 11 (deletion size: 7–20 Mb, with a breakpoint at 11q23.3 in 70 to 80% of cases) (2). Over 200 cases have been reported to date, with a female:male ratio of 2:1.

Jacobsen syndrome encompasses a broad spectrum of clinical phenotypes (**Supplementary Figure 1**). About half of the patients are diagnosed in the first 12 months of life, whereas individuals with milder, less obvious signs and symptoms may be diagnosed at an older age (3). The most frequently present clinical features include facial and skeletal anomalies, cognitive impairment, and congenital malformations of the central nervous system, heart, urogenital tract, and gastrointestinal tract. Some patients also present thrombocytopenia, impaired platelet function, and endocrine, ocular or hearing disorders.

To date, immunologic abnormalities have been reported in 43 patients with Jacobsen syndrome (4–19). While 35 patients had abnormal immunoglobulin (Ig) levels (IgG, IgA, or IgM), only 17 had low IgG levels (6–9, 14–16) and some of the patients were receiving Ig replacement therapy. A low total B cell count was found in 28 patients, and seven had low numbers of switched memory B cells (CD27+IgD-) (6, 9), suggesting a defective germinal center function. Poor antibody responses to vaccines in some patients show further evidence that Jacobsen syndrome is not only associated with B-cell, but also T-cell dysfunction. A more detailed immunological evaluation of twelve patients with Jacobsen syndrome was reported by Baronio et al., showing that all the main lymphocyte compartments, including T cells, show alterations in cell differentiation (15). In-depth analyses of T and B cell phenotypes in Jacobsen syndrome and their correlation to deletions of individual genes located in the 11q region are scarce.

The following immune-related genes are located within the part of chromosome 11 that is potentially deleted in patients with Jacobsen syndrome (9): erythroblast transformation-specific 1 (*ETS1*), Toll/interleukin-1 receptor domain-containing adapter protein (*TIRAP*), Friend leukemia integration 1 (*FLI1*), nuclear factor related to kappa B binding protein (*NFRKB*), thymocyte nuclear protein 1 (*THYN1*), and sorting nexin 19 (*SNX19*). Lymphopenia, low B cell, T cell and natural killer (NK) subpopulation counts, and inadequate T cell responses to mitogens or antigens have variously been reported in Jacobsen syndrome (4–16), but there is disagreement on the correlation between immunodeficiency and deleted genes on 11q like *ETS1*.

Here, we describe the immunologic phenotypes and the deletion of immune-related genes in four patients with Jacobsen syndrome followed up at our institution.

## Case description

Four patients with Jacobsen syndrome were followed up between 2010 and 2021 in the Division of Immunology at the University Children’s Hospital Zurich (Zurich, Switzerland). The patients were enrolled in an ongoing study of monogenic diseases. Written informed consent was provided by each patient and/or his/her legal guardian. The study was approved by the hospital’s institutional review board (reference: PB_2016_02280) and was registered at ClinicalTrials.gov (NCT02735824).

**Figure 1:**
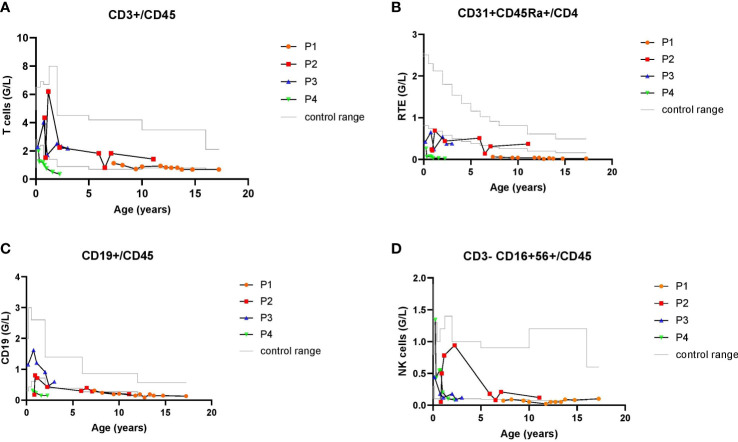
Longitudinal lymphocyte values of P1-4 with Jacobsen syndrome during their follow-up between 2 and 10 years: total T cells counts **(A)**, recent thymic emigrants, RTE **(B)**, CD19+ B cells **(C)** and CD16+CD56+ NK cells **(D)**. *Gray lines delineate age-specific upper and lower reference values of the cell counts*.

The oldest patient (P1) is now a 17-year-old male who was diagnosed with Jacobsen syndrome at the age of 7 years. A 6.4 Mb deletion (11q24.2 to 11q25) was found (**Supplementary Table 1**). He had typical facial features, cognitive impairment, a ventricular septal defect (VSD), which closed spontaneously, mild caliectasis of the renal pelvis (which also regressed spontaneously), and abnormal platelet function (a surface receptor defect). Regarding immunologic features, P1’s medical history showed no recurrent infections other than occasional rhinitis. The immunologic work-up revealed mild hypogammaglobulinemia (IgG, IgG1, IgA, and IgM; **Supplementary Table 2**). Although the anti-tetanus antibodies were adequate, antibodies against pneumococci were absent. However, P1 had never been vaccinated against pneumococci. He presented with mild lymphocytopenia and low numbers of naive T cells, and recent thymic emigrants (RTEs) (**Figure 1**). It is noteworthy that the patient’s total T cell count had been normal during preschool age, but deteriorated over time. The NK- and B-cell counts (non-switched and switched B cells) were low. Upon T cell stimulation with mitogen (phorbol myristate acetate (PMA), staphylococcal enterotoxin B (SEB), and phytohemagglutinin (PHA)), CD69 expression was normal. An overview of the patient’s immunological features in synopsis with the patients reported in the literature is given in **Supplementary Table 2**.

P2 is an 11-year-old girl with partial Jacobsen syndrome, the genetic diagnosis of which was established at the age of 3 years. A 9.8 Mb deletion (11q21 to 11q22.3) was found. She had dysmorphic facial features, cognitive impairment, a small atrial septum defect, and a horseshoe kidney that required pyeloplasty (due to outlet stenosis). The hematological work-up showed normal platelet function. At 1 year of age, P2 became ill with a pneumococcal pleuropneumonia; the course of disease was severe, and she required mechanical ventilation for 6 days and drainage of pleural effusion. After recovery from this infection, she developed a prolonged episode of fever with typical clinical features of Kawasaki syndrome, including dilatation of the left coronary artery. The patient was treated with acetylsalicylic acid and intravenous Ig, which rapidly resolved the fever. However, five days after the intravenous Ig infusion, P2’s general condition deteriorated again, and she developed fever and hepatosplenomegaly. Several specific laboratory findings were noted, including anemia, thrombocytopenia, elevated ferritin, hypofibrinogenemia, and hemophagocytosis documented after a bone marrow aspirate. Overall, the patient’s clinical and laboratory features met the diagnostic criteria for hemophagocytic lymphohistiocytosis (HLH). The patient’s condition improved markedly after treatment with methylprednisolone. Primary HLH was ruled out by an immunologic, functional, and genetic work-up. After recovery from the inflammatory episode and after treatment had ceased, the patient developed alopecia universalis. Her immunologic results revealed mild B cell lymphopenia but otherwise normal lymphocyte counts. Naive T cell count and RTEs were normal, except during episodes of stress (such as during pneumonia and HLH at the age of 1 year and after heart surgery at the age of 6, cf. **Figure 1** and **Supplementary Table 2**).

P3 is a 5-year-old girl who was diagnosed with Jacobsen syndrome immediately after birth. Molecular karyotyping showed a 15.1 Mb deletion (11q23 to 11qter). She had typical dysmorphic facial features, a perimembranous VSD, hexadactyly, and cognitive impairment. Although the platelet aggregation test was abnormal, P3 did not have an abnormal bleeding tendency. Her medical history showed recurrent pulmonary infections, most of which (except for a severe episode of bacterial pneumonia) were viral (**Supplementary Table 1**). Her immunologic work-up showed a low RTE count (**Figure 1** and **Supplementary Table 2**), otherwise normal T cell counts, normal levels of immunoglobulins and anti-vaccine antibody titers, normal B- and NK-cell counts, and a normal T cell stimulation response after exposure to mitogens.

P4 is a 2-year-old girl who was diagnosed with Jacobsen syndrome immediately after birth, following the observation of intrauterine growth retardation and congenital heart malformations (aortic isthmus stenosis, aortic arch hypoplasia, and malalignment VSD). Molecular karyotyping showed a 12.9 Mb deletion (11q24.1 to 11q25). Other *de novo* problems were subsequently noted: short stature, underweight, cognitive impairment, and abnormal platelet function. Although P4 did not suffer from recurrent infections, immunologic monitoring revealed a progressive decline in IgG, IgG1, and IgM levels. The patient had adequate anti-vaccine antibody titers against tetanus and pneumococci, although the anti-*H. influenzae* antibody titer fell swiftly. P4 developed lymphopenia, with a very low total T cell count and low numbers of naive T cells and RTEs (**Figure 1**). Her B cell counts had started to fall in first few months of life. The NK cell count remained within the normal range. T cell marker expression after stimulation with mitogens (PMA, SEB and PHA) was also normal. Despite an uneventful clinical course of P4, who was kept in home isolation by her parents, the significant deterioration in immunologic parameters prompted us to administer immunoglobulin replacement therapy and cotrimoxazole prophylaxis from the age of 21 months onwards. Lastly, P4 has not been given any live vaccines.

To establish whether the patients’ immunodeficiencies might have an association with the 11q genotype, we determined which immune-related genes were located in the deleted segment of chromosome 11. Chromosomal microarray analysis using an Affymetrix Cytoscan HD array with 2.65 million probes at a 20 kb resolution was used for all 4 patients. Whole exome sequencing (WES) was not performed in our cohort. We found that the *ETS1* gene had been deleted in the three patients (P1, P3 and P4) with low RTE counts and low non-switched memory B cell (CD27+IgD+) counts. Only P1 and P4 showed additional T and B cell abnormalities. *TIRAP, FLI1, NFRK, THYN1*, and *SNX19* were located in the 11q deletion found in P3 and P4.

## Discussion

Here, we describe the immunologic and genetic features of four patients with Jacobsen syndrome monitored at our institution. All four showed some peculiar immunologic features. Although none had experienced recurrent bacterial infections, two suffered from an episode of severe bacterial pneumonia and another had been on antibiotic prophylaxis in early childhood. Only one patient showed recurrent viral infections. One of the patients showed signs of immune dysregulation, with Kawasaki syndrome and secondary HLH. Hypogammaglobulinemia was observed in two patients, and low total B cell counts with variable, low titers of vaccine-specific antibodies were observed in two patients. Low numbers of RTEs were found in three patients, two of whom also had overall T cell lymphopenia.

All three patients with a low RTE count had heterozygous loss of *ETS1*, whereas the patient with a normal (but borderline) RTE count did not. The low numbers of RTEs could not be attributed to surgical removal of the thymus, since one of the three patients had undergone cardiac surgery with preservation of the thymic tissue and the other two had not undergone cardiac surgery. Lastly, none of the patients with a low RTE count had been treated with steroids (another possible factor influencing the number of RTEs).

The low RTE counts in our patients with Jacobsen syndrome were always associated with low numbers of non-switched memory B cells, which is consistent with a low thymic output and a functional T cell impairment. One of the patients with loss of *ETS1* (P3) did not show any other immunologic abnormalities apart from the low numbers of RTEs and non-switched memory B cells. However, this patient is currently only 5 years old and so the subsequent development of other immunologic abnormalities cannot be ruled out. In two patients, immunologic variables deteriorated with age. Delayed onset of increased susceptibility to infections and immune abnormalities in older patients with variable immune deficiency phenotype has been described previously in patients with Jacobsen syndrome (4, 9, 17).

*ETS1* is a strong candidate for the T cell defect in Jacobsen syndrome (4, 10, 12–14, 20). A growing body of evidence shows that erythroblast transformation specific (ETS) factors are essential immune system regulators. Nine of the 29 known ETS factors might regulate genes involved in immunity, including ETS1, ETS2, GABP, FLI1, ELF1, MEF, ESE1, PU.1, and SpiB (21). *ETS1* knockout and hypomorphic mice show aberrant thymic differentiation, abnormally low peripheral T cell numbers, an abnormal Th1 immune response, and aberrant Th17 differentiation (22–24).

Evidence on T cell abnormalities, especially RTEs, and deletion in *ETS1* is scarce. There is only one description of a 16-year-old patient with Jacobsen syndrome with recurrent infections and low RTEs where genetic investigation on gene *ETS1* was performed. In this patient, *ETS1* was deleted (4). A more recent publication of a 46-year-old patient with recurrent infections showed deletion of *ETS1* and *FLI1*. Low T cell counts were seen, but no investigation on RTEs was performed (14). A cohort of 12 pediatric patients with profound lymphocyte alteration revealed low RTEs in all patients. But in this cohort no genetic investigation on *ETS1* was reported (15). On the other hand, there is a report of a 26-year-old patient with Jacobsen syndrome without any T-cell abnormality in spite of a loss of *ETS1* (17). Likewise, a more recent report of a cohort comprising 14 patients with 11q deletion did not find any correlation of immunodeficiency of B or T lymphocytes and *ETS1* or *FLI1* (16). In both investigations, RTEs were not assessed (16, 17).

In addition to *ETS1* deletion, we found heterozygous deletions of *TIRAP, FLI1, NFRKB, THYN1*, and *SNX19* in two of our patients*. TIRAP* codes for an adapter protein involved in the Toll-like receptor (TLR)2 and TLR4 innate immune response signaling pathways (25). *FLI1* encodes a transcription factor containing an ETS DNA-binding domain. Heterozygous deletion of *FLI1* may lead to a lack of T helper cells and a low serum IgM level (5). *NFRKB* encodes the DNA-binding protein R-κB, which regulates IL2R α-chain gene expression and is essential for T cell activation (13). *THYN1*, encoding a thymocyte nuclear protein found in CD34+ hematopoietic stem and progenitor cells, may have a role in apoptosis (12). *SNX19* is involved in intracellular vesicle trafficking and exocytosis (26). The patients with a heterozygous deletion of all six immune-related genes showed different immunologic phenotypes. One of the patients already had a profound primary immunodeficiency at a very early age, with hypogammaglobulinemia, a decreasing anti-*Haemophilus influenza* antibody titer, a decreasing total B cell count, and low numbers of non-switched memory B cells, total T cells, naive T cells, and RTEs. The other patient with heterozygous deletion of all the 6 immune-related genes had experienced a severe bacterial infection and displayed low numbers of non-switched memory B cells and RTEs but no other severe immunologic changes. Although heterozygous gene deletions offer an explanation for these findings, additional *de novo* imbalances in the patient with the most profound immunologic abnormalities might have contributed to the phenotype.

In one patient with severe immune dysregulation with Kawasaki syndrome and secondary HLH, who later developed alopecia universalis, none of the six immune-related genes was deleted. Immune dysregulation might either have occurred sporadically in this patient, without any genetic predisposition for immune-related disorders, or hitherto not yet described genetic abnormalities could be responsible for the immunological phenotype in this patient.

In conclusion, our data support the notion that Jacobsen syndrome is associated with a combined immunodeficiency with variable presentation. The genetic causes of the T and B cell defects in our four patients remain elusive. Although *ETS1* might be a promising candidate for T cell abnormalities in patients with Jacobsen syndrome, genotype-phenotype analyses of larger cohorts are warranted. The variable phenotype-genotype may be due to incomplete penetrance, but also due to other genes of interest located on 11q such as *TIRAP, FLI1, NFRKB, THYN1*, and *SNX19*, together with other as-yet poorly characterized genes in the deleted segment (27). It could be hypothesized that a whole gene cluster is involved in cell proliferation and differentiation and could play a role in the development of the combined immunodeficiency in 11q patients. Genes of interest that might act as modifier are ATM, CD3, and CBL, which are also located on chromosome 11. Moreover, sequence variants in genes involved in other inborn errors of immunity may contribute to the clinical phenotype in patients with interstitial or terminal deletions of 11q.

The results of our case series emphasize the importance of immunologic follow-up in all patients with Jacobsen syndrome, since immune abnormalities and greater susceptibility to infections may develop over time. Further investigations of putative genetic predictors of immunodeficiency in patients with Jacobsen syndrome are required.

## Data availability statement

Publicly available datasets were analyzed in this study. This data can be found here: www.deciphergenomics.org, DECIPHER accession numbers: 368156, 456461, 456462, 456463.

## Ethics statement

This study was reviewed and approved by cantonal ethics committee Zurich, Switzerland. Written informed consent to participate in this study was provided by the participants’ legal guardian/next of kin. Written informed consent was obtained from the individual(s), and minor(s)’ legal guardian/next of kin, for the publication of any potentially identifiable images or data included in this article.

## Author contributions

JS, SP, TT were the clinicians in charge of patient care and management. TT extracted the data. TT, JS, SP, KS contributed to the drafting and revision of the case report. All authors contributed to the article and approved the submitted version.

## Funding

This work was funded by the Clinical Research Priority Program CYTIMM-Z (University of Zurich, Switzerland) and the Swiss National Science Foundation grant 320030_205097 (Berne, Switzerland) to JS.

## Acknowledgments

We thank the patients and their families for their kind cooperation and the participating healthcare staff for their support. We thank Dr. David Fraser (Biotech Communication SARL, Ploudalmézeau, France) for copy-editing assistance and Dina Pitts for technical assistance.

## Conflict of interest

The authors declare that the research was conducted in the absence of any commercial or financial relationships that could be construed as a potential conflict of interest.

## Publisher’s note

All claims expressed in this article are solely those of the authors and do not necessarily represent those of their affiliated organizations, or those of the publisher, the editors and the reviewers. Any product that may be evaluated in this article, or claim that may be made by its manufacturer, is not guaranteed or endorsed by the publisher.
