# Supplementary material for: Case report: ETS1 gene deletion associated with a low number of recent thymic emigrants in three patients with Jacobsen syndrome
Source: Front Immunol. 2022 Oct 21;13:867206. doi: 10.3389/fimmu.2022.867206 (PMC9634179; doi:10.3389/fimmu.2022.867206)
Supplement: Supplementary file 1 [file Image_1.pdf]

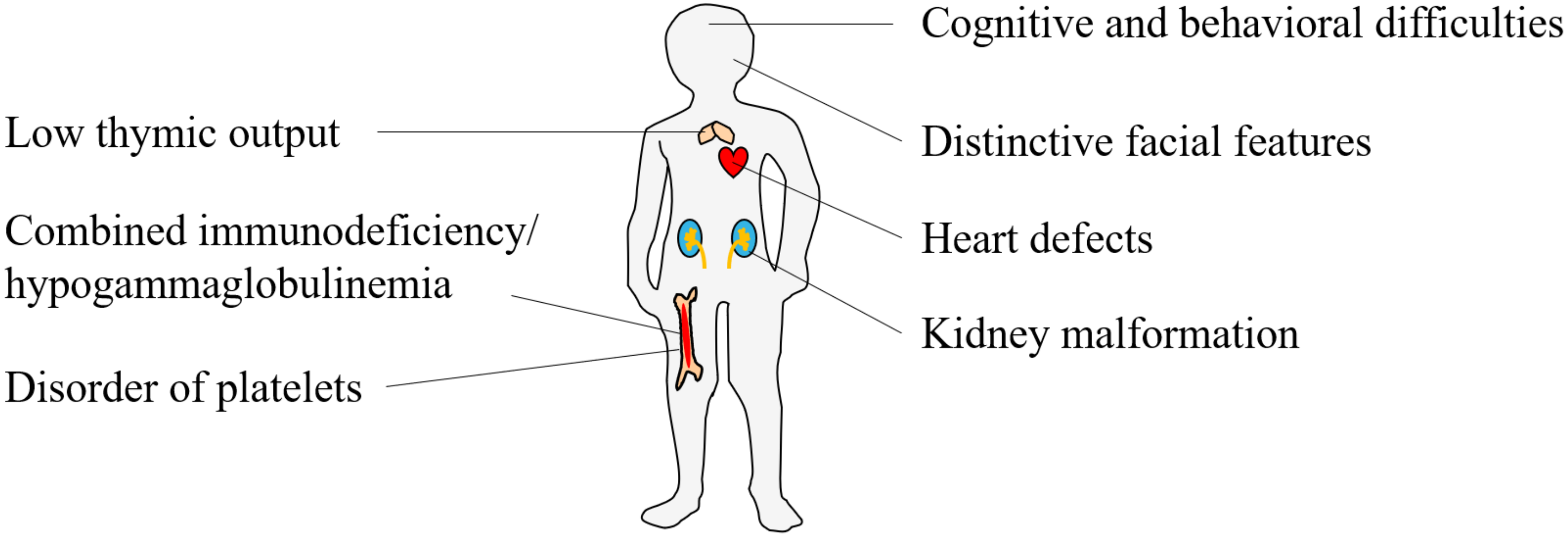

Supplementary Figure 1: Clinical Features of Jacobsen Syndrome. Common clinical findings are shown by affected organ system.
