# Supplementary material for: Case report: ETS1 gene deletion associated with a low number of recent thymic emigrants in three patients with Jacobsen syndrome
Source: Front Immunol. 2022 Oct 21;13:867206. doi: 10.3389/fimmu.2022.867206 (PMC9634179; doi:10.3389/fimmu.2022.867206)
Supplement: Supplementary file 2 [file Table_1.pdf]

Supplementary Table 1. Phenotype and genotype of four patients with Jacobsen syndrome

	P1	P2	P3	P4
Sex [m=male, f=female]	m	f	f	f
Age [years]	17	11	5	2
Diagnosis	Jacobsen syndrome	partial Jacobsen syndrome	Jacobsen syndrome	Jacobsen syndrome
**Genetics**	11q24.2–11q25	11q21–11q22.3	11q23–qter	11q24.1–11q25, additional *de novo* imbalances
Deletion size	6.4 Mb	9.8 Mb	15.1 Mb	12.9 Mb
Notable deleted genes	ETS1	none	TIRAP, ETS1, FLI1, NFRKB, THYN1, SNX19	TIRAP, ETS1, FLI1, NFRKB, THYN1, SNX19
**Clinical phenotype**				
Facial dysmorphism	yes	yes	yes	yes
Developmental delay	yes	yes	yes	yes
Cardiac defect	yes	yes	yes	yes
	ventricular septal defect (closed spontaneously)	atrial septal defect	perimembranous ventricular septal defect	aortic isthmus stenosis, aortic arch hypoplasia, ventricular septal defect
Abnormal platelet function	yes	no	yes	yes
	surface receptor defect		abnormal platelet aggregation test	not specified
Other features	mild caliectasis of the renal pelvis	horseshoe kidney with outlet stenosis	hexadactyly	
**Immune-related conditions**				
Recurrent infections				
viral	rare (rhinitis)	rare, mild	yes	very rare
bacterial	no	yes (pulmonary)	yes (pulmonary)	no
Hospital admission for severe infections	no	yes (pneumococcal pleuropneumonia)	yes	no
Immune dysregulation:	none	Kawasaki syndrome, secondary hemophagocytic lymphohistiocytosis, alopecia universalis	none	none
**Treatment**				
Intravenous immunoglobulins (IvIg)	no	1x IvIg therapy (for the treatment of Kawasaki syndrome)	no	yes (from the age of 21 months)
Pneumocystis jirovecii prophylaxis	no	no	no	yes

Supplementary Table 2. Overview immunodeficiency in patients with Jacobsen syndrome

Patient resp. Reference	Age [years]	Sex [f/m]	Recurrent infections [yes/no]	IgA [g/l]	IgM [g/l]	IgG [g/l]	IgG Anti-tetanus toxin [U/ l]	IgG Anti-Häm. infl. Typ B [µg/ml]	IgG Anti-S. Pneumoniae [µg/ml]	Leukocytes [Giga/L]	Lymphocytes [Giga/L]	CD3+/CD45 [Giga/L]	CD3+CD4+CD8-/CD45 [Giga/L]	CD3+CD8+CD4-/CD45 [Giga/L]	CD4/CD8 Ratio	CD4+CD45RA+/CD3 [Giga/L]	CD31+CD45Ra+/CD4 [Giga/L]	CD19+/CD45 [Giga/L]	CD27+IgD-/CD20 [%]	CD3-CD16+56+/CD45 [Giga/L]	Affected gene: ETS1	
**P1**	17	m	no	0.41	0.18	6.12	268.00	NA	<0.5	4.95	0.95	0.69	0.41	0.23	1.80	0.13	0.02	0.13	3	0.10	yes	
**P2**	11	f	no	1.81	0.66	13.58	<100	NA	8.11	4.15	1.75	1.42	0.88	0.40	2.20	0.47	0.37	0.19	8	0.12	no	
**P3**	3	f	yes	0.83	0.38	13.90	749.00	0.97	2.51	7.41	2.99	2.18	1.35	0.72	1.90	1.05	0.38	0.60	4	0.12	yes	
**P4**	2	f	no	0.12	0.09	2.71	1226.00	<0.15	4.00	2.95	0.78	0.51	0.40	0.08	5.00	0.16	0.03	0.15	2	0.10	yes	
**(16)**	40[a]	f	yes	1.93	0.22	9.1*	NA	NA	NA	8.70	0.68	0.58	0.49	0.09	5.44	NA	NA	0.04	NA	0.09	yes	~
**(16)**	10[b]	f	yes	0.82	<0.5	12.2*	NA	NA	NA	2.80	0.63	0.55	0.32	0.23	1.39	NA	NA	0.04	NA	0.03	yes	~
**(16)**	7	m	yes	0.40	0.13	3.92*	NA	NA	NA	6.60	2.90	0.11	0.07	0.04	1.75	NA	NA	0.14	NA	0.08	yes	~
**(16)**	1	f	no	0.31	0.20	6.80	NA	NA	NA	6.20	2.71	1.99	1.28	0.65	1.97	NA	NA	0.83	NA	0.17	yes	~
**(16)**	0	f	no	0.26	0.14	3.80	NA	NA	NA	6.20	3.62	1.71	1.17	0.50	2.34	NA	NA	0.50	NA	0.29	yes	~
**(16)**	4	f	yes	1.40	0.30	11.30	NA	NA	NA	4.90	1.76	1.27	0.62	0.60	1.03	NA	NA	0.46	NA	0.10	yes	~
**(16)**	15[c]	f	yes	0.91	0.24	17.9*	NA	NA	NA	3.20	0.95	0.85	0.44	0.30	1.47	NA	NA	0.09	NA	0.13	yes	~
**(16)**	4	m	yes	0.96	0.44	7.70	NA	NA	NA	6.10	0.90	0.58	0.22	0.32	0.69	NA	NA	0.15	NA	0.14	yes	~
**(16)**	8	f	yes	1.59	0.45	8.10	NA	NA	NA	6.00	0.76	0.63	0.27	0.35	0.77	NA	NA	0.17	NA	0.06	yes	~
**(16)**	22	f	yes	2.07	0.55	10.50	NA	NA	NA	5.50	1.63	1.23	0.69	0.48	1.44	NA	NA	0.17	NA	0.19	no	
**(16)**	16[d]	m	yes	0.47	0.21	0.8*	NA	NA	NA	6.70	1.63	1.14	0.74	0.38	1.95	NA	NA	0.21	NA	0.40	no	
**(16)**	6	m	yes	0.41	0.30	8.00	NA	NA	NA	13.30	3.84	3.12	2.27	0.79	2.87	NA	NA	0.64	NA	0.26	no	
**(16)**	16	f	yes	1.83	1.28	11.70	NA	NA	NA	5.80	2.16	1.67	0.89	0.68	1.31	NA	NA	0.35	NA	0.44	no	
**(16)**	18	f	yes	0.65	0.97	5.2*	NA	NA	NA	17.50	2.08	1.53	1.06	0.47	2.26	NA	NA	0.11	NA	0.46	no	
**(14)**	46	m	yes	0.56	0.15	1.54	NA	NA	NA	8.60	0.41	0.42	0.27	0.15	1.80	NA	NA	0.00	NA	0.07	yes	
**(15)**	0.4	NA	no	<0.06	<0.28	1.28	NA	NA	NA	2.30	0.90	0.33	0.21	0.12	1.79	0.05	0.02	0.27	0.70	0.26	NA	
**(15)**	0.6	NA	no	0.53	0.33	4.00	NA	NA	NA	7.40	3.70	3.13	1.88	1.06	1.77	1.52	0.59	0.37	2.00	0.36	NA	
**(15)**	1	NA	yes	0.20	0.34	5.52	NA	NA	NA	3.94	1.30	0.81	0.40	0.34	1.16	0.16	0.05	0.37	2.20	0.18	NA	
**(15)**	4	NA	yes	0.53	0.44	8.03	NA	NA	NA	5.30	2.00	1.05	0.77	0.25	3.13	0.59	0.09	0.22	11.60	0.20	NA	
**(15)**	4	NA	yes	0.45	0.32	4.50	NA	NA	NA	4.40	1.60	1.27	0.61	0.60	1.01	0.41	0.16	0.19	1.60	0.13	NA	
**(15)**	5	NA	yes	0.45	0.10	4.07	NA	NA	NA	3.50	0.80	0.32	0.24	0.07	3.72	NA	NA	0.09	12.80	0.07	NA	
**(15)**	6	NA	yes	0.39	0.08	4.43	NA	NA	NA	2.70	1.20	0.93	0.43	0.47	0.92	0.26	0.09	0.75	2.80	0.12	NA	
**(15)**	6	NA	yes	0.20	0.33	8.41	NA	NA	NA	4.90	1.40	0.89	0.43	0.38	1.12	0.03	0.00	0.15	2.30	0.33	NA	

**(15)**	12	NA	no	0.74	0.53	12.83	NA	NA	NA	4.50	0.90	0.63	0.21	0.41	0.51	0.07	0.02	0.05	4.50	0.20	NA	
**(15)**	16	NA	yes	1.40	0.42	9.02	NA	NA	NA	2.90	1.10	1.00	0.43	0.53	0.81	0.12	0.46	0.05	5.10	0.05	NA	
**(15)**	20	NA	yes	0.48	1.20	8.40	NA	NA	NA	9.60	2.90	2.20	1.32	0.76	1.73	0.74	NA	0.33	5.00	0.38	NA	
**(15)**	22	NA	no	0.46	0.70	9.77	NA	NA	NA	6.30	1.90	1.81	0.95	0.74	1.28	0.43	NA	0.11	9.00	0.11	NA	
**(12)**	6	m	yes	normal	0.40	normal	NA	NA	NA	1.50	0.60	0.60	<0.3	<0.3	NA	NA	NA	0.10	NA	NA	yes	#
**(17)**	26	m	yes	normal	normal	normal	NA	NA	NA	NA	NA	normal	NA	NA	NA	NA	NA	0.08	NA	normal	yes	
**(11)**	2	f	no	normal	normal	normal	NA	NA	NA	NA	NA	low	NA	NA	NA	NA	NA	low	NA	low	NA	
**(11)**	0.1	f	NA	low	normal	normal	NA	NA	NA	NA	low	low	NA	NA	NA	NA	NA	low	NA	normal	NA	
**(18)**	16	f	yes	NA	low	NA	NA	NA	NA	NA	NA	NA	NA	NA	NA	NA	NA	NA	NA	NA	NA	¢
**(4)**	16	f	yes	0.53	<0.17	8.91	NA	NA	NA	NA	NA	0.76	0.31	0.40	0.78	0.11	0.05	0.04	NA	0.18	yes	§
**(6)**	24	m	yes	0.45	<0.3	5.80	NA	NA	low	NA	NA	0.82	0.20	0.60	0.33	NA	NA	0.10	low	0.04	NA	
**(6)**	35[a]	f	no	1.89	<0.3	7.50	NA	NA	normal	NA	NA	0.64	0.50	0.10	5.00	NA	NA	0.05	low	0.08	NA	
**(6)**	14	f	yes	0.65	0.97	5.20	NA	NA	low	NA	NA	1.38	0.90	0.40	2.25	NA	NA	0.22	normal	0.23	NA	
**(6)**	14[d]	m	yes	0.30	<0.3	3.10	NA	NA	low	NA	NA	1.18	0.70	0.40	1.75	NA	NA	0.16	low	0.18	NA	
**(6)**	6[c]	f	yes	0.88	0.17	3.20	NA	NA	low	NA	NA	0.77	0.40	0.30	1.33	NA	NA	0.09	low	0.04	NA	
**(6)**	10[b]	f	yes	0.61	<0.3	3.50	NA	NA	low	NA	NA	0.48	0.30	0.20	1.50	NA	NA	0.07	low	0.04	NA	
**(9)**	45	f	yes	0.66	0.40	3.40	0.07	NA	low	2.10	0.55	0.50	0.17	0.33	0.50	NA	NA	0.02	low	0.05	NA	
**(7)**	12	m	yes	0.31	0.06	2.26	normal	low	low	NA	0.90	0.72	0.46	0.30	1.54	NA	NA	0.06	NA	0.07	NA	
**(19)**	10	m	yes	normal	low	normal	NA	NA	low	NA	NA	NA	NA	NA	NA	NA	NA	NA	NA	NA	NA	
**(8)**	4	m	yes	0.17	0.15	2.81	NA	normal	low	NA	NA	normal	NA	NA	NA	NA	NA	normal	NA	NA	NA	
**(8)**	5	m	yes	0.19	0.25	1.61	normal	NA	low	NA	low	NA	NA	NA	NA	NA	NA	NA	NA	NA	NA	
**(5)**	34	m	yes	NA	0.35	NA	NA	NA	NA	NA	NA	NA	0.20	NA	NA	NA	NA	NA	NA	normal	NA	
**(13)**	3	f	yes	normal	normal	high	NA	NA	NA	NA	1.70	1.30	0.48	0.76	0.63	NA	NA	0.30	NA	0.18	yes	
**(10)**	12	f	yes	0.28	0.15	5.40	NA	NA	NA	3.00	0.72	NA	NA	NA	NA	NA	NA	NA	NA	NA	yes	°

Note: Numbers in red indicate pathologically low values, numbers in blue indicate pathologically high values.

Abbreviations: P1, patient 1; P2, patient 2; P3, patient 3; P4, patient 4

* *During immunoglobulin replacement therapy*
a-d *Corresponding patients investigated in the two publications*

other genes of interest:
~ *FLI1*
# *FLI1, JAM3, THYN1*
¢ *TIRAP*
§ *FLI1, TIRAP, NFRKB, THYN1, SNX19*
° *FLI1, NFRKB*
